# Graphene Surface Reinforcement of Iron

**DOI:** 10.3390/nano9010059

**Published:** 2019-01-04

**Authors:** Pengjie Wang, Qiang Cao, Yuping Yan, Yangtian Nie, Sheng Liu, Qing Peng

**Affiliations:** 1 The Institute of Technological Sciences, Wuhan University, Wuhan 430072, China; pjwang@whu.edu.cn (P.W.); nieyangtian@whu.edu.cn (Y.N.); 2 School of Power and Mechanical Engineering, Wuhan University, Wuhan 430072, China; yypgoodluck87@163.com (Y.Y.); victor_liu63@126.com (S.L.); 3 Nuclear Engineering and Radiological Sciences, University of Michigan, Ann Arbor, MI 48109, USA

**Keywords:** nanoindentation, graphene/Fe composite, critical yield strength, hardness, elastic modulus

## Abstract

Graphene is an ideal material in the reinforcement of metal-matrix composites owing to its outstanding mechanical and physical properties. Herein, we have investigated the surface enhancement of iron via a computational nanoindentation process using molecular dynamics simulations. The findings of our study show that graphene can enhance the critical yield strength, hardness and elastic modulus of the composite to different degrees with the change of the number of graphene layers. In the six tested models, the composite with trilayer graphene on the surface produces the strongest reinforcement, with an increased magnitude of 432.1% and 169.5% in the hardness and elastic modulus, respectively, compared with pure iron. Furthermore, it is revealed that high temperature could weaken the elastic bearing capacity of the graphene, resulting in a decrease on the elastic mechanical properties of the graphene/Fe composite.

## 1. Introduction

Since its discovery [1], graphene has been the most attractive material to be explored because of its remarkable electronic and physical properties due to the quantum confinement [2,3,4,5]. In particular, graphene has shown great potential in matrix reinforcement in recent years owing to its outstanding strength over 1 TPa [5]. In general, a low modulus matrix can be significantly reinforced by the presence of high-modulus graphene, which is called filler in composites [6]. The common assumption that the filler modulus is independent of the matrix has been proved incorrect, considering the wide range of reinforcement on polymer matrices by high-modulus graphene [6]. Many factors influence the mechanical properties of graphene-based nanocomposites, including the structure of the filler, the synthetic method of the composite, the concentration of the filler in the matrix, the interactions between the filler and the matrix, and the orientation of the filler. Even with a very small amount of graphene, the composite’s Young’s modulus, tensile strength and toughness can have sharp increases [7,8].

Extensive studies have been conducted on metal matrix composites, including Fe, Al, Cu, Mg and Ni [9,10,11,12,13]. Iron is by far the most commonly used industrial metal on account of its great range of desirable properties and low cost. Dislocations play an important role in revealing the remarkable mechanical properties of iron matrix composite, about which a punch-out mechanism has been proposed to explain the formation of interstitial dislocation loops [14]. The study on the interaction between edge dislocations and graphene nanosheets in graphene/Fe composites by molecular dynamics (MD) simulations revealed an increase of 107% and 1400% in shear modulus and yield stress, respectively [9]. Meanwhile, the enhancement of surface hardness on iron is another crucial issue in broad industrial applications of graphene. Graphene is an excellent choice for the surface reinforcement of pure iron matrix due to the relatively simple industrial process, the study on which can offer guidance to the manufacture and application of the graphene/Fe composite. However, relevant efforts have been rarely reported.

Nanoindentation is an approach widely used to measure elastic modulus and hardness of nanocomposites [15,16]. In this study we investigated the enhancement of hardness and elastic modulus of iron by graphene additives. The amount of graphene measured by the number of layers was explicitly examined using nanoindentation modeling. We recorded the load-displacement data during the nanoindentation process of the graphene/Fe composite made by a diamond indenter. By analyzing the load-displacement curves, we compared the hardness and elastic modulus between the pure iron matrix and composites with graphene on the surface and in the superficial zone. In addition, the influence of different loading speeds and temperatures on the elastic mechanical properties of the composite were also discussed.

## 2. Method

### 2.1. Model

**Figure 1:**
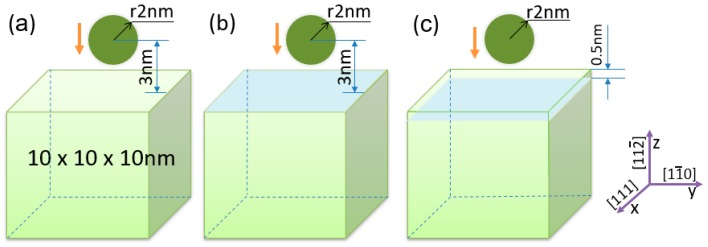
(**a**) Simulation cell of pure iron matrix; (**b**) simulation cell of composite with graphene on the surface of the matrix, the graphene could be monolayer, bilayer and trilayer; (**c**) simulation cell of composite with graphene in the superficial zone of the matrix, the graphene could be monolayer, bilayer and trilayer.

The MD method was used to examine the influence of graphene in the graphene/Fe composite at the atomic level. The large-scale atomic/molecular massively parallel simulator (LAMMPS) software was employed to calculate the MD simulations. The simulation cells are shown in Figure 1. The x and y directions are set the periodic boundary condition and the z direction is fixed.

The graphene/Fe composite models are shown in Figure 1b,c. In this study the BCC α-Fe matrix is an area of 10 × 10 × 10 nm with a lattice constant of 2.85 Å, which is shown in Figure 1a. Figure 1b shows the composite model with graphene on the surface of the matrix. The composite with graphene in the superficial zone of the matrix is shown in Figure 1c, where the distance between the graphene layer and the top surface is 0.5 nm. All balls with a radius of 2 nm consisting of carbon atoms in diamond structure have the same velocity moving down to make a nanoindentation on the surface of the composite or pure iron matrix.

### 2.2. Molecular Dynamics Simulations

The accuracy of the MD simulation results is determined by the selection of potential function. We used the Brenner-generation reactive empirical bond-order potential to model the C-C bonded interaction [17]. The C-C AIREBO potential has a widespread application in graphene-based materials [18,19], which is composed of three terms:

(1) $$E=\frac{1}{2}\sum_{i}\sum_{j\neq i}\left[E_{ij}^{REBO}+E_{ij}^{LJ}+\sum_{k\neq i,j}\sum_{l\neq i,j,k}E_{kijl}^{TORSION}\right],$$

where the *E_ij_^REBO^* term describes the short-ranged interactions (r < 2 Å) between carbon atoms, the E*_ij_^LJ^* term adds longer-ranged interactions (2 Å < r < cutoff) using a form similar to the standard Lennard Jones potential, and the E*_kijl_^TORSION^* term describes various dihedral angle preferences in hydrocarbon configurations, which is an explicit 4-body potential.

The embedded-atom method (EAM) potential was used to compute pairwise interactions between iron atoms [20]. The total energy *E_i_* of an atom *i* is specified as

(2) $$E_{i}=F_{\alpha }\left(\sum_{j\neq i}\rho_{\beta }\left(r_{ij}\right)\right)+\frac{1}{2}\sum_{j\neq i}\emptyset_{\alpha \beta }\left(r_{ij}\right),$$

where *F_α_* is the embedding energy which is a function of the atomic electron density *ρ_β_*. *Φ_αβ_* is the pair potential interaction between atoms *I* and *J*, as a function of the distance *r_ij_* between atom *I* and atom *J*. α and β are atomic element types. The C-Fe interaction between iron and carbon atoms of both diamond indenter and graphene layers was described by the classical Lennard Jones (LJ) potential, as shown in Equation (3)

(3) $$E=4\varepsilon \left[{\left(\frac{\sigma }{r}\right)}^{12}-{\left(\frac{\sigma }{r}\right)}^{12}\right]\;r<r_{c},$$

where *r_c_* is the cutoff distance. The *σ* and *ε* for the C-Fe interaction are 2.221 Å and 0.043 eV [21], respectively. The LJ potential was also used to model the interaction between the graphene layers and diamond indenter with the σ and ε value of 3.4 Å and 0.00284 eV [22], respectively.

We set a downward velocity of 30 m/s on the balls and kept the temperature at 300 K by the Nose-Hoover algorithm [23]. After the energy minimization, the downward movement would continue until a preset depth and then the ball moved upward. The timestep was 0.001 ps. The displacement and the force in the z-direction of the diamond indenter imposed by the composite matrix were recorded.

### 2.3. Nanoindentation

The Hertzian contact analysis theory guided our model. To understand the influence of graphene sheets on the hardness and elastic modulus of the iron matrix, the load-displacement data was recorded for analysis. The load-displacement relation is described by [24]

(4) $$P=\frac{4}{3}\sqrt{R}E_{r}{\left(h-h_{f}\right)}^{\frac{3}{2}},$$

where P is the z-direction force of diamond indenter, h is the max depth of the nanoindentation, h_f_ is the final displacement of the plastic unloading process. R is defined by *R* = (1/*R*_1_ + 1/*R*_2_)^−1^, where R_1_ is the radius of diamond indenter, R_2_ is the radius of the spherical hole in the surface of the substrate [24]. The induced modulus E_r_ is given by [25]

(5) $$\frac{1}{E_{r}}=\frac{\left(1-\nu^{2}\right)}{E}+\frac{\left(1-\nu_{i}^{2}\right)}{E_{i}},$$

where *E* and *ν* are Elastic modulus (Young’s modulus) and Poisson’s ratio of the composite matrix, *E_i_* and *ν_i_* are the same parameters of the diamond indenter. The hardness is defined by

(6) $$H=\frac{P_{max}}{A_{c}},$$

where *P_max_* is the max force at the initial unloading point, and *A_c_* is the contact area of spherically curved surface [25].

## 3. Results

### 3.1. Monolayer Graphene Enhancement

**Figure 2:**
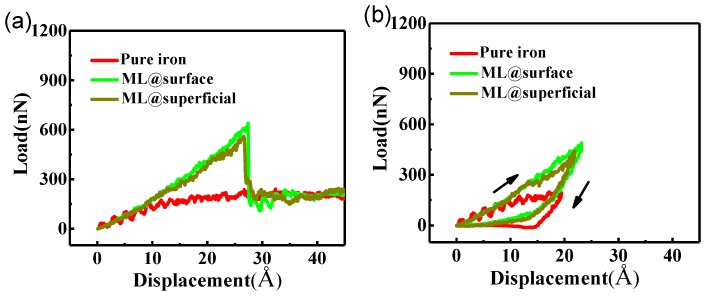

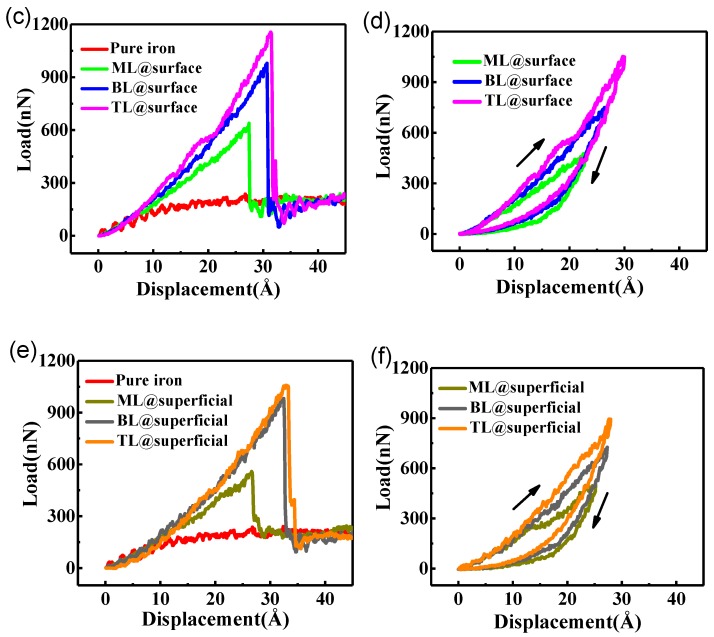
(**a**) Load-displacement curves of pure iron, composite with monolayer (ML) graphene on surface and in superficial zone; (**b**) loading-unloading curves of pure iron and composite with monolayer graphene; (**c**) load-displacement curves of pure iron and composite with different numbers of layers on the surface (ML, bilayer (BL), trilayer (TL)); (**d**) loading-unloading curves of three surface composite cases; (**e**) load-displacement curves of pure iron and composite with different number layers in superficial zone; (**f**) loading-unloading curves of three superficial composite cases.

The results of the simulated nanoindentation process are revealed by the load-displacement curves. Figure 2a shows the load-displacement curves of the three cases, i.e., pure iron, monolayer graphene on the surface and monolayer graphene in the superficial zone of the graphene/Fe composite. The max displacement of the diamond indenter is 4.5 nm. These results indicate that the monolayer graphene has a significant reinforcement on iron matrix both in surface and superficial zone cases. In the initial 1 nm displacement, the load of the three cases shares almost the same growth rate, indicating that graphene has no evident effect on iron matrix in the early stage of loading. After that, the pure iron loading curve gradually levels off, while the curves of the other two composites keep growing. The difference in curves indicates that graphene acts as a strong deterrent for the yield of the composite. From Figure 2a, we can see that the graphene has increased the yield strength of the composites obviously. The sharp drop points of the curves indicate that the load bearing capability of the graphene has reached its maximum and then the graphene is fracted. After that, the load curves of the three cases begin to converge. We made the loading-unloading processes of those three cases in their plastic deformation stages. The load change of the three cases is shown in Figure 2b. The arrows indicate the loading and unloading processes. The serration in the curve of pure iron is mainly related to the potential we adopted to describe the C-Fe interaction. The elastic modulus and hardness of pure iron and the two composite cases with monolayer graphene were calculated according to the loading-unloading curves. The hardness of pure iron is 8.1, very close to the experimental value of 8.2 [26]. The elastic modulus of pure iron is 150.1 GPa, a little lower than the experimental value of 200 GPa [26] and a MD result in shear modulus of 56.4 GPa [9], which is associated with the potentials used in the simulation. The two parameters of the composite with monolayer graphene on the surface are 19.4 and 218.4 GPa, an increase of 139.5% and 45.5%, respectively, compared with the pure iron case. In the case of composites with graphene in the superficial zone, the hardness and elastic modulus are 18.4 and 201.1 GPa, an increase of 127.2% and 33.3%, respectively. In general, the monolayer graphene has greatly increased the hardness and elastic modulus of the composite due to its remarkable load bearing capability. Meanwhile, the composite with graphene on the surface produces a better reinforcement. The local stress and interspace would be produced as the graphene embedded into the iron matrix, which enhances the flexural rigidity and weakens the elastic bearing capacity of the graphene, resulting in a decrease of the hardness and elastic modulus.

### 3.2. Multilayer Graphene Enhancement

**Figure 3:**
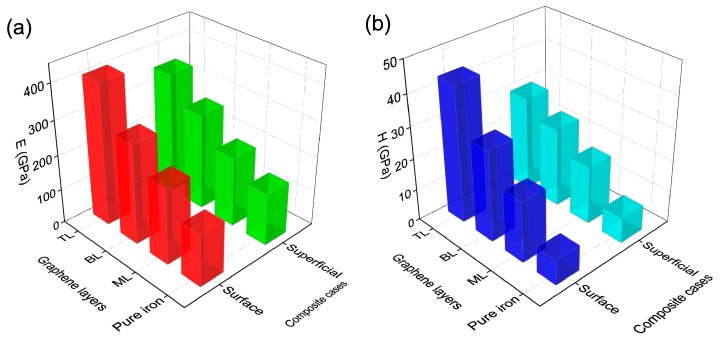
(**a**) Elastic modulus of composite cases (pure iron, ML, BL, TL); (**b**) hardness of composite cases.

The mechanical properties of multiple graphene have received growing interest in recent years [27,28]. To investigate the reinforcement of graphene/Fe composites with different numbers of graphene layers, we explored monolayer, bilayer and trilayer graphene on the surface and in the superficial zone. Figure 2c shows the loading processes of the composite with different numbers of graphene layers on the surface. It was observed that the critical yield strength of the composite increases with the number of graphene layers, due to the improvement of the load bearing capability by adding more graphene layers. The critical nanoindentation depth is also extended from 2.7 to 3.2 nm, owing to the enhancement of elastic deformation capacity of the composite, as the graphene increases from monolayer to trilayer. Figure 2d shows the loading-unloading curves of the three surface cases. The hardness and elastic modulus of composite with bilayer graphene on the surface shown in Figure 3 are 28.9 and 286.5 GPa, with increases of 256.8% and 90.9%, respectively, compared with the pure iron case. The same parameters of composite with trilayer graphene on the surface are 43.1 and 404.5 GPa, corresponding to increases of 432.1% and 169.5%, respectively, compared with the pure iron case. The results suggest that the graphene layers have an effective improvement on the elastic mechanical properties of the iron matrix. With the increase of graphene layers, the max load increases significantly while the contact area changes slightly, leading to the proportional increase of hardness and elastic modulus. Figure 2e shows the loading processes of the composites with graphene in the superficial zone. The relationship between yield strength and graphene layers is similar to the surface cases. However, the max yield strength of bilayer graphene case is only a little smaller than the trilayer case in the superficial zone cases. In the superficial trilayer case, the local stress was produced on the top layer and under the bottom layer graphene region, which caused the rugged surfaces under the top graphene layer and on the bottom graphene layer. The middle layer graphene appeared to slip due to the unbalanced van der Waals force from the other two graphene layers at the initial time. The degree of distortion on the middle graphene layer is uneven during the nanoindentation process, which weakens the loading bearing capacity in a way. As a result, the max yield strength of trilayer case did not improve much more than the bilayer case. The loading-unloading curves of the three cases in superficial zone are shown in Figure 2f. Figure 3 also shows the hardness and elastic modulus of composite with different number layers graphene in superficial zone. The hardness and elastic modulus are 24.8 and 275.0 GPa for the bilayer case, 29.6 and 347.1 GPa for the trilayer case. The increments of those two parameters are 206.2% and 83.2% for bilayer case, 265.4% and 131.2% for the trilayer case, compared with the pure iron case. The variation tendency on elastic modulus and hardness of all the surface and superficial cases are shown in Figure 3a,b, respectively. The two parameters of composite in surface cases are higher than that in superficial zone cases to different degrees with the same number of graphene layers, which is related to the local stress fields produced by lattice mismatch between the graphene and iron matrix in the superficial situations. The stress fields reduce the flexibility of graphene layers and lower the critical yield strength of the graphene/Fe composite.

### 3.3. Effect of Loading Speed and Temperature

**Figure 4:**
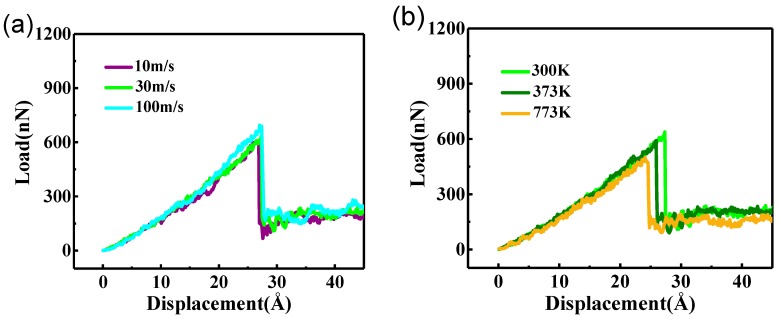
(**a**) Load-displacement curves of composite with monolayer graphene on surface in different loading speeds; (**b**) load-displacement curves of composite with monolayer graphene on surface at different temperatures.

To investigate the influence of loading speed and temperature on the nanoindentation process of the graphene/Fe composite, we explored the nanoindentation simulation of the composite with monolayer graphene on surface at varied loading speeds and at different temperatures, as shown in Figure 4a,b, respectively. It can be seen that the critical yield strength in the case with a loading speed of 100 m/s is a little higher than the other two cases, which suggests that the change of loading speed has a slight effect on the loading bearing capacity of the graphene. The situation for 10 m/s resembles the 30 m/s case, and all the speed cases have almost the same critical nanoindentation depth, which reveals that the elastic bearing capacity of the composite has no relationship with the loading speed. Meanwhile, the effect of different temperatures is more obvious. The critical yield strength and nanoindentation depth decreased significantly with the increase of temperature, which is related to the effect on the crystal texture of the iron matrix and graphene by different temperatures. As the temperature increases, the lattice vibration of graphene becomes intensified, which greatly reduces its mechanical properties, resulting in a weakening of elastic bearing capacity [29]. Meanwhile, the high temperature improves the atomic activity, lowers the grain boundary strength and reduces the lattice resistance in the iron matrix region, which also has a negative effect on the loading bearing capacity of the composite.

The simulation results reveal the change of hardness and elastic modulus of iron matrix composites with the graphene of the different number of layers, which is well consistent with previous studies on metal-matrix composites reinforced by graphene [30,31]. Both the surface and the superficial composite cases have a significant reinforcement on the iron matrix to different degrees. In our models, the composite with trilayer graphene on the surface has the strongest hardness of 43.1 GPa and the maximum elastic modulus of 404.5 GPa, corresponding to an increase of 432.1% and 169.5%, respectively, compared to the pure iron case. Further studies could be conducted to optimize the results, such as the size of the diamond indenter, potential functions and the system size. Furthermore, the reinforcement caused by the graphene in other morphologies in the iron matrix is also expected to be further explored.

## 4. Conclusions

To conclude, we have investigated the mechanical properties change of graphene/Fe composites with different number of graphene layers on the surface and in the superficial zone by using the MD simulation method to model the nanoindentation process. The results from the loading-displacement curves show that the graphene layers have a significant effect on the critical yield strength, hardness and elastic modulus of iron matrix in different degrees due to its remarkable loading bearing capacity. The degree of reinforcement on iron matrix increases with the number of graphene layers, both in surface and superficial zone cases. It was demonstrated that the enhancement in hardness and elastic modulus of composite in the surface cases is slightly stronger than that in superficial zone cases with graphene of the same number of layers on account of the production of the local stress fields in the superficial situations. Finally, the loading speeds have a small effect on the simulation of the composite nanoindentation process, and the simulation results of different temperatures reveal that high temperature can effectively reduce the mechanical properties of the graphene/Fe composite by softening lattice rigidity.
